# Supplementary material for: Joint Degeneration in a Mouse Model of Pseudoachondroplasia: ER Stress, Inflammation, and Block of Autophagy
Source: Int J Mol Sci. 2021 Aug 26;22(17):9239. doi: 10.3390/ijms22179239 (PMC8431545; doi:10.3390/ijms22179239)
Supplement: Supplementary file 1 [file ijms-22-09239-s001.zip › ijms-1299565-supplementary.pdf]

International Journal of *Molecular Sciences*

Supplemental Figure S1. Absence of mutant-COMP expression correlates with no articular chondrocyte stress in MT-COMP mice.

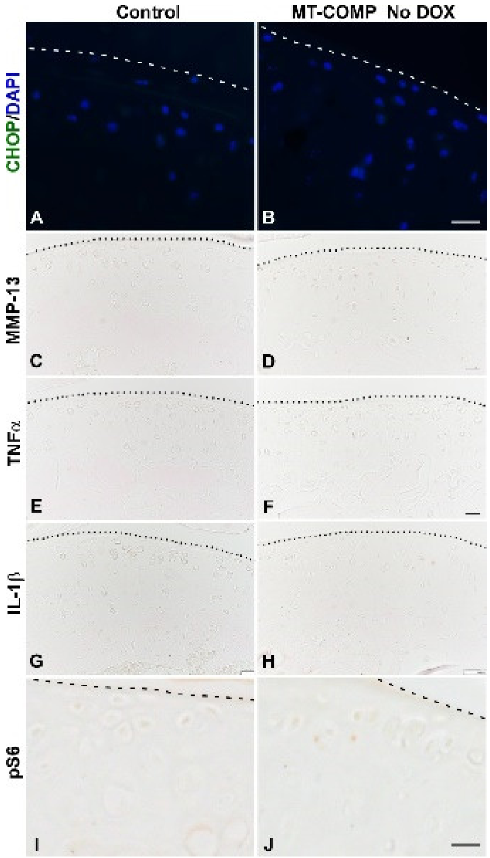

Supplemental Figure S1. Absence of mutant-COMP expression correlates with no articular chondrocyte stress in MT-COMP mice. Dotted line marks the edge of articular cartilage. Articular cartilage from control and MT-COMP mice with no DOX at 20 weeks were immunostained for CHOP (green, **A**, **B**) and DAPI (blue nuclei, **A**, **B**) or MMP13 (brown **C**, **D**) or TNFα (brown **E**, **F**) or IL-1β (brown **G**, **H**) or pS6 (brown **I**, **J**). ER stress (CHOP), degradative enzyme (MMP13), inflammation (TNFα or IL-1β), and block of autophagy (pS6) were not detected in either MT-COMP or control articular chondrocytes in the absence of DOX that induce mutant-COMP expression. Bar = 100 μm.
